# A guide to selecting high-performing antibodies for ARID1B (UniProt ID: Q8NFD5) for use in western blot and immunoprecipitation

**DOI:** 10.12688/f1000research.179398.1

**Published:** 2026-04-28

**Authors:** Vera Ruíz Moleón, Sara González Bolívar, Riham Ayoubi, Vincent Francis, Peter S. McPherson, Carl Laflamme

**Affiliations:** 1 Department of Neurology and Neurosurgery, Structural Genomics Consortium, The Montreal Neurological Institute, McGill University, Montreal, Québec, Canada

**Keywords:** Q8NFD5, ARID1B, ARID1B, BAF250B antibody characterization, antibody validation, western blot, immunoprecipitation

## Abstract

ARID1B encodes a core subunit of the SWI/SNF (BAF) ATP-dependent chromatin-remodeling complex and plays a critical role in transcriptional regulation, cell differentiation, and development. Here we have characterized seven ARID1B commercial antibodies for western blot and immunoprecipitation by using a standardized experimental protocol based on comparing read-outs in knockout cell lines and isogenic parental controls. These studies are part of a larger, collaborative initiative seeking to address antibody reproducibility issues by characterizing commercially available antibodies for human proteins and publishing the results openly as a resource for the scientific community. While the use of antibodies and protocols vary between laboratories, we encourage readers to use this report as a guide to select the most appropriate antibodies for their specific needs.

## Introduction

ARID1B is a key component of the SWI/SNF (BAF) chromatin-remodeling complex and plays an essential role in transcriptional regulation, cellular differentiation, and development. ^1^ By modulating chromatin accessibility, ARID1B controls gene expression programs critical for neurodevelopment and tissue homeostasis. Germline pathogenic variants in ARID1B are a major cause of Coffin–Siris syndrome and related neurodevelopmental disorders, while somatic alterations have been identified in a wide range of malignancies, supporting its role as a tumor suppressor. ^2, 3^

This research is part of a broader collaborative initiative in which academics, funders and commercial antibody manufacturers are working together to address antibody reproducibility issues by characterizing commercial antibodies for human proteins using standardized protocols, and openly sharing the data. ^4^ It consists of identifying human cell lines with adequate target protein expression and the development/contribution of equivalent knockout (KO) cell lines, followed by antibody characterization procedures using most commercially available renewable antibodies against the corresponding protein. ^4^ Here we characterized seven commercial ARID1B antibodies, selected and donated by participant antibody manufacturers, for use in western blot and immunoprecipitation (also referred to as immunocytochemistry), enabling biochemical and cellular assessment of ARID1B properties and function.

The authors do not engage in result analysis or offer explicit antibody recommendations. Our primary aim is to deliver top-tier data to the scientific community, grounded in Open Science principles. This empowers experts to interpret the characterization data independently, enabling them to make informed choices regarding the most suitable antibodies for their specific experimental needs. Guidelines on how to interpret antibody characterization data found in this study are featured on the YCharOS gateway ^5^ and in Table 4 of this data note. ^4^

**Table 1.** Summary of the cell lines used.

Institution	Catalog number	RRID (Cellosaurus)	Cell line	Genotype
Horizon discovery	C631	CVCL_Y019	HAP1	WT
Horizon discovery	HZGHC000582c007	CVCL_SD45	HAP1	*ARID1B* KO
Abcam	ab255451	CVCL_0291	HCT 116	WT
Abcam	ab287219	CVCL_B8BG	HCT 116	*ARID1B* KO

**Table 2.** Summary of the ARID1B antibodies tested.

Company	Catalog number	Lot number	RRID (Antibody registry)	Clonality	Clone ID	Host	Concentration (μg/μL)	Vendors recommended applications
Abcam	ab57461 *	1123625–2	AB_2243092	Monoclonal	2D2	Mouse	0.50	Wb, IF
Abcam	ab300619 **	GR3452889–3	AB_2886323	Recombinant mono	EPR25408–34	Rabbit	0.49	Wb, IF
Cell signalling	65747 **	1	AB_2799694	Recombinant mono	E1U7D	Rabbit	0.02	Wb, IP
Cell signalling	92964 **	3	AB_2810599	Recombinant mono	E9J4T	Rabbit	0.20	Wb, IP
GeneTex	GTX130708	42081	AB_2886326	Polyclonal		Rabbit	1.32	Wb
Millipore sigma	MABN2266 *	Q2966662	NA	Monoclonal	3D9.1	Mouse	0.50	Wb, IF
Proteintech	82979–1-RR **	23007015	AB_3670727	Recombinant mono	230222G4	Rabbit	1.00	IF

Wb = western blot; IF = immunofluorescence; IP = immunoprecipitation,

** = recombinant antibody,

* = monoclonal antibody, NA = not available

**Table 3.** Table of secondary antibodies used.

Company	Secondary antibody	Catalog number	RRID (Antibody registry)	Clonality	Concentration (μg/μL)	Working concentration (μg/mL)
Proteintech	HRP-Goat Anti-Rabbit Antibody (H + L)	RGAR001	AB_3073505	Recombinant polyclonal	1.0	0.05
Proteintech	HRP-Goat Anti-Mouse Antibody (H + L)	RGAM001	AB_3068333	Recombinant polyclonal	1.0	0.5
MilliporeSigma	Protein A, HRP conjugate	18–160	NA	Polyclonal	1.0	5.0
Abcam	VeriBlot for IP Detection Reagent (HRP)	ab131366	AB_2892718	Polyclonal	0.04	0.08

**Table 4.** Illustrations to assess antibody performance in western blot and immunoprecipitation.

Western blot	Immunoprecipitation
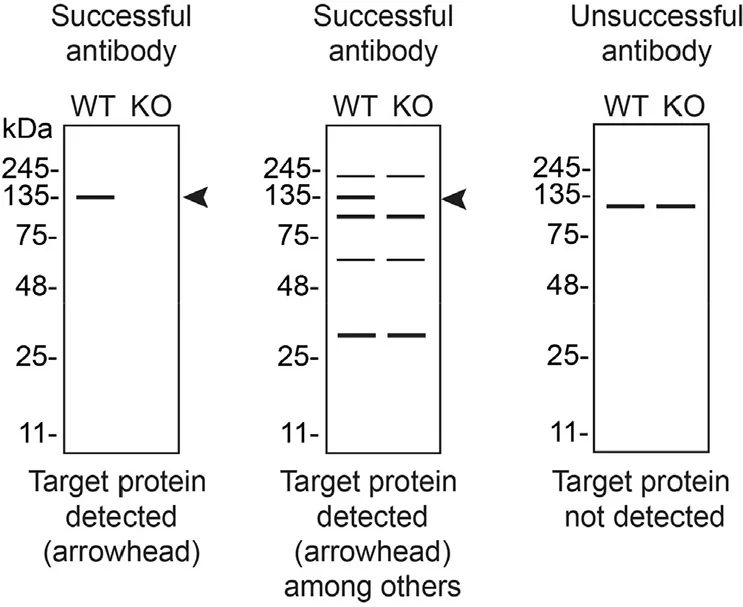	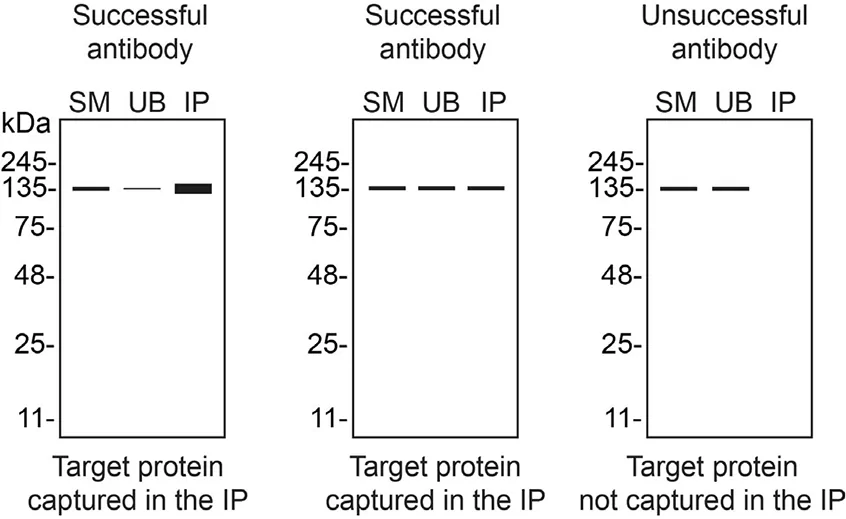

This table has been reproduced with permission from Ayoubi et al., Elife, 2023 ^8^

## Results and discussion

Our standard protocol involves comparing readouts from wild type (WT) and KO cells. ^6, 7^ The first step was to identify a cell line(s) that expresses sufficient levels of a given protein to generate a measurable signal using antibodies. To this end, we examined the DepMap (Cancer Dependency Map Portal, RRID: SCR_017655) transcriptomics database to identify all cell lines that express the target at levels greater than 2.5 log _2_ (transcripts per million “TPM” + 1), which we have found to be a suitable cut-off. ^8^ The HAP1 and HCT 116 cell lines express the *ARID1B* transcript at 3.7 and 4.3 log _2_ TPM + 1. A *ARID1B* KO HAP1 cell line and *ARID1B* KO HCT 116 cell line was obtained from Horizon Discovery and Abcam respectively ( Table 1). Moreover, as seen on DepMap, the cell lines HAP1 and HCT 116 do not carry mutations in the *ARID1B* that could affect antibody–epitope binding.

**Figure 1.**
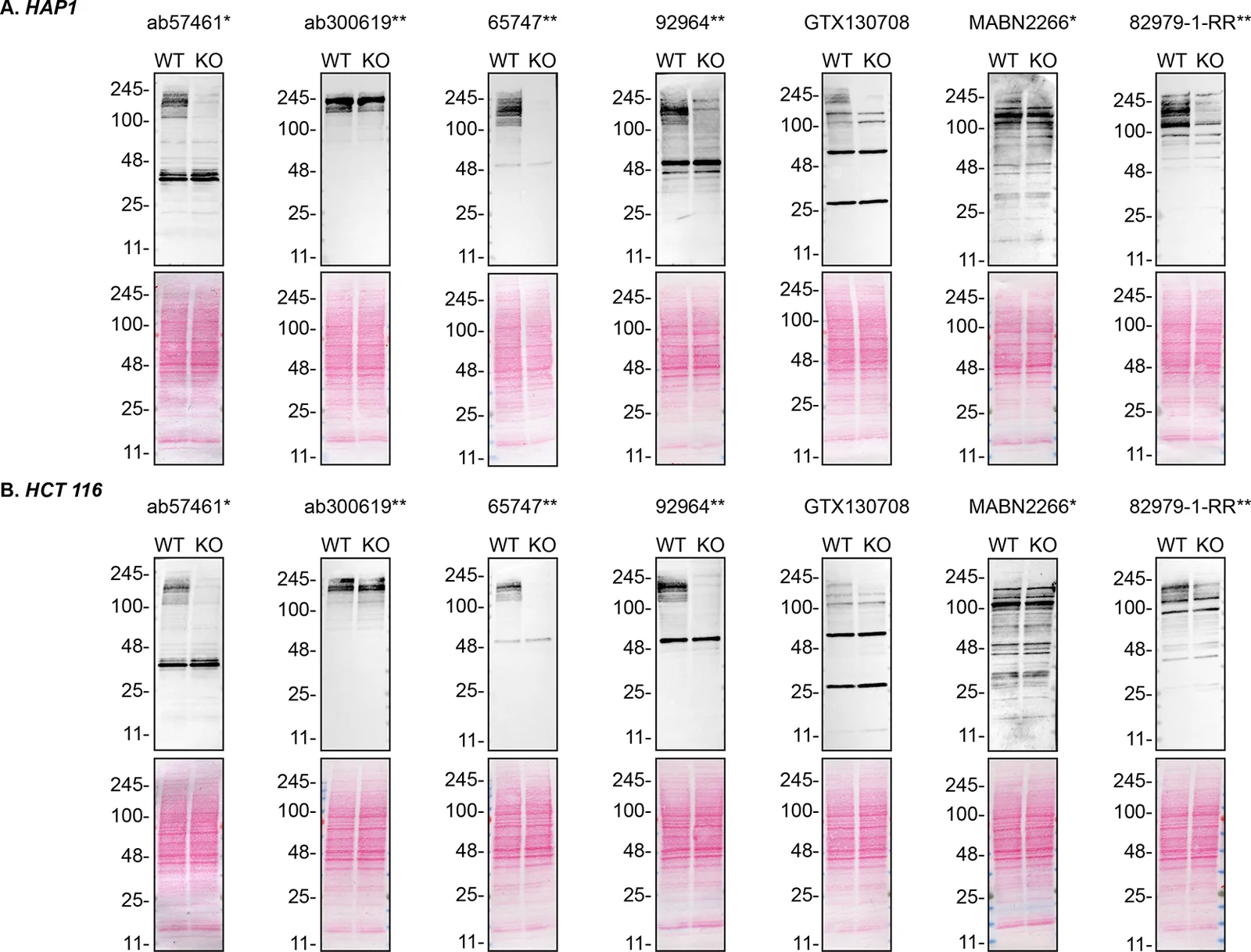
ARID1B antibody screening by western blot. Lysates of **A)** HAP1 and **B)** HCT 116 (WT and *ARID1B* KO) were prepared, and 30 μg of protein were processed for western blot with the indicated ARID1B antibodies. The Ponceau stained transfers of each blot are presented to show equal loading of WT and KO lysates and protein transfer efficiency from the acrylamide gels to the nitrocellulose membrane. Antibody dilutions were chosen according to the recommendations of the antibody supplier. Antibody dilutions used: ab57461* at 1/1000; ab300619** at 1/1000; 65747** at 1/200; 92964** at 1/200; GTX130708 at 1/500; MABN2266* at 1/1000; 82979–1-RR** at 1/1000. Predicted band size: 243 kDa. ** = recombinant antibody, * = monoclonal antibody.

To screen all seven by western blot, WT and *ARID1B* KO protein lysates from both lines were ran on SDS-PAGE, transferred onto nitrocellulose membranes, and then probed with the seven ARID1B antibodies in parallel ( Figure 1).

**Figure 2.**
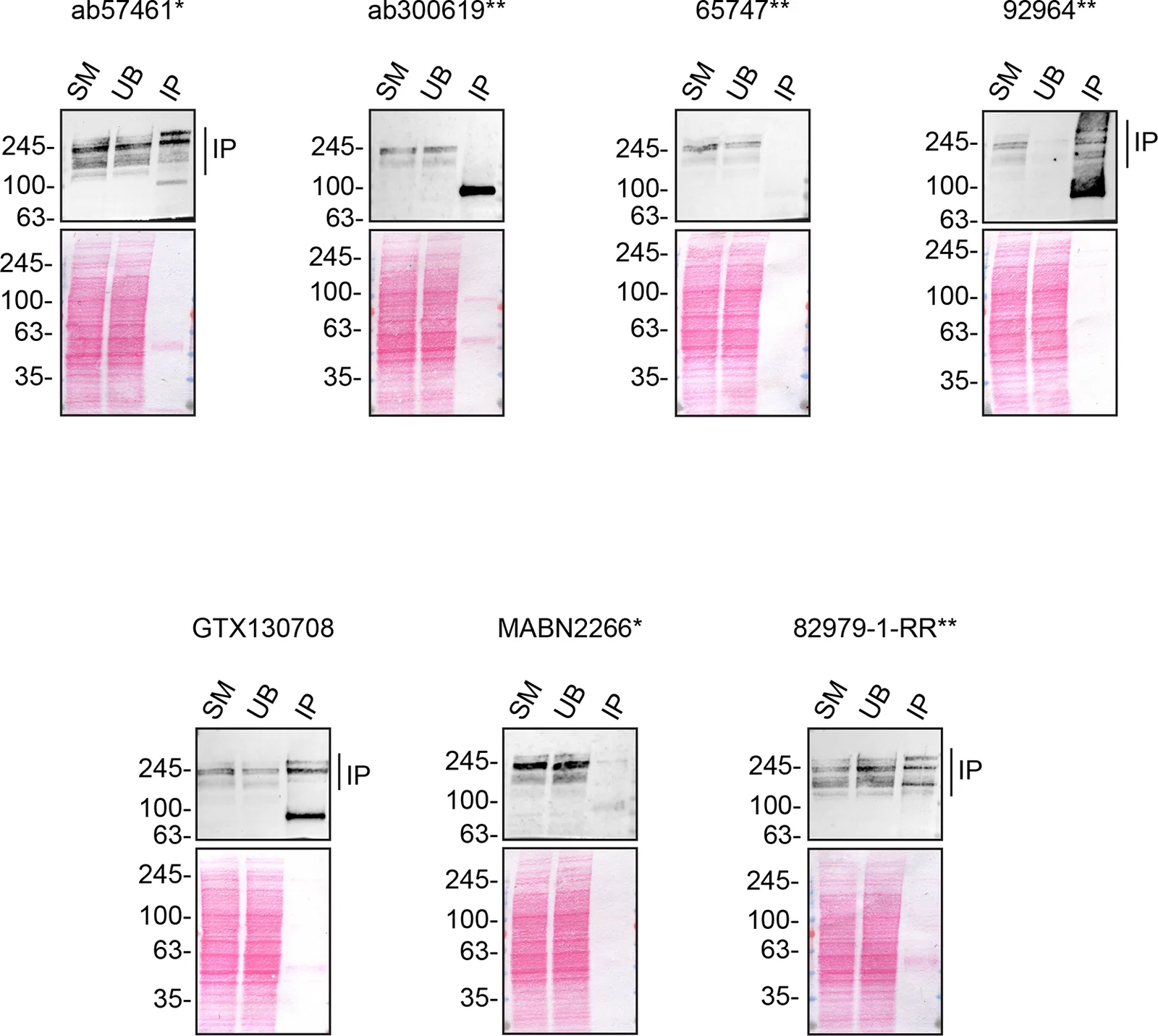
ARID1B antibody screening by immunoprecipitation. HAP1 WT lysates were prepared, and immunoprecipitation was performed for 1 h using 0.5 mg of lysate and 2.0 μg of the indicated ARID1B antibodies pre-coupled to Dynabeads protein A or protein G. Samples were washed and processed for western blot with the anti-ARID1B 65747** used at 1/200. The Ponceau stained transfers of each blot are shown. SM = 6% starting material; UB = 6% unbound fraction; IP = immunoprecipitate. ** = recombinant antibody, * = monoclonal antibody.

We then assessed the capability of all seven antibodies to capture ARID1B from HAP1 protein extracts using immunoprecipitation techniques, followed by western blot analysis. For the immunoblot step, a specific ARID1B antibody identified previously (refer to Figure 1) was selected. Equal amounts of the starting material (SM) and the unbound fractions (UB), as well as the whole immunoprecipitate (IP) eluates were separated by SDS-PAGE ( Figure 2).

In conclusion, we have screened seven ARID1B commercial antibodies by western blot, and immunoprecipitation by comparing the signal produced by the antibodies in human HAP1 and HCT 116 (WT and *ARID1B* KO) cells. To assist users in interpreting antibody performanyce, Table 4 outlines various scenarios in which antibodies may perform in both applications. ^8^ High-quality and renewable antibodies that successfully detect ARID1B were identified for western blot and immunoprecipitation. Researchers who wish to study and in a different species are encouraged to select high-quality antibodies, based on the results of this study, and investigate the predicted species reactivity of the manufacturer before extending their research.

### Limitations

Inherent limitations are associated with the antibody characterization platform used in this study. Firstly, the YCharOS project focuses on renewable (recombinant and monoclonal) antibodies and does not test all commercially available ARID1B antibodies. YCharOS partners provide approximately 80% of all renewable antibodies, but some top-cited polyclonal antibodies may not be available through these partners. We encourage readers to consult vendor documentation to identify the specific antigen each antibody is raised against, where such information is available.

Secondly, the YCharOS effort employs a non-biased approach that is agnostic to the protein for which antibodies have been characterized. The aim is to provide objective data on antibody performance without preconceived notions about how antibodies should perform or the molecular weight that should be observed in western blot. As the authors are not experts in ARID1B, only a brief overview of the protein’s function and its relevance in disease is provided. ARID1B experts are invited to analyze and interpret observed banding patterns in western blots.

Thirdly, YCharOS experiments are not performed in replicates primarily due to the use of multiple antibodies targeting various epitopes. Once a specific antibody is identified, it validates the protein expression of the intended target in the selected cell line, confirms the lack of protein expression in the KO cell line and supports conclusions regarding the specificity of the other antibodies. All experiments are performed using master mixes, and meticulous attention is paid to sample preparation and experimental execution. In IF, the use of two different concentrations serves to evaluate antibody specificity and can aid in assessing assay reliability. In instances where antibodies yield no signal, a repeat experiment is conducted following titration. Additionally, our independent data is performed subsequently to the antibody manufacturers internal validation process, therefore making our characterization process a repeat.

Lastly, as comprehensive and standardized procedures are respected, any conclusions remain confined to the experimental conditions and cell line used for this study. The use of a single cell type for evaluating antibody performance poses as a limitation, as factors such as target protein abundance significantly impact results. Additionally, the use of cancer cell lines containing gene mutations poses a potential challenge, as these mutations may be within the epitope coding sequence or other regions of the gene responsible for the intended target. Such alterations can impact the binding affinity of antibodies. This represents an inherent limitation of any approach that employs cancer cell lines.

## Method

The standardized protocols used to carry out this KO cell line-based antibody characterization platform was established and approved by a collaborative group of academics, industry researchers and antibody manufacturers. The detailed materials and step-by-step protocols used to characterize antibodies in western blot, immunoprecipitation and immunofluorescence are openly available on Protocols.io ( protocols.io/view/a-consensus-platform-for-antibody-characterization ). ^4^ Brief descriptions of the experimental setup used to carry out this study can be found below.

### Cell lines and antibodies

The cell lines, primary and secondary antibodies used in this study are listed in Table 1, 2, and 3, respectively. To ensure consistency with manufacturer recommendations and account for proprietary formulations (where antibody concentrations are not disclosed), antibody usage is reported as dilution ratios rather than absolute concentrations. To facilitate proper citation and unambiguous identification, all cell lines and antibodies are referenced with their corresponding Research Resource Identifiers (RRIDs). ^9, 10^ All cell lines used in this study were regularly tested for mycoplasma contamination and were confirmed to be mycoplasma-free.

### Antibody screening by western blot

HAP1 and HCT 116 (WT and *ARID1B* KO) cells were collected in RIPA buffer (25mM Tris-HCl pH 7.6, 150mM NaCl, 1% NP-40, 1% sodium deoxycholate, 0.1% SDS) (Thermo Fisher Scientific, cat. number 89901) supplemented with 1x protease inhibitor cocktail mix (MilliporeSigma, cat. number P8340). Lysates were sonicated briefly and incubated 30 min on ice. Lysates were spun at ~110,000 *x g* for 15 min at 4°C and equal protein aliquots of the supernatants were analyzed by SDS-PAGE and western blot. BLUelf prestained protein ladder (GeneDireX, cat. number PM008-0500) was used.

Western blots were performed with precast midi 4-20% Tris-Glycine polyacrylamide gels (Thermo Fisher Scientific, cat. number WXP42012BOX) ran with Tris/Glycine/SDS buffer (Bio-Rad, cat. number 1610772), loaded in Laemmli loading sample buffer (Thermo Fisher Scientific, cat. number AAJ61337AD) and transferred on nitrocellulose membranes. Proteins on the blots were visualized with Ponceau S staining (Thermo Fisher Scientific, cat. number BP103-10) which is scanned to show together with individual western blot. Blots were blocked with 5% milk for 1 hr, and antibodies were incubated O/N at 4°C with 5% milk in TBS with 0,1% Tween 20 (TBST) (Cell Signalling Technology, cat. number 9997). Following three washes with TBST, the peroxidase conjugated secondary antibody was incubated at a dilution of ~0.2 μg/ml in TBST with 5% milk for 1 hr at room temperature followed by three washes with TBST. Membranes were incubated with Pierce ECL (Thermo Fisher Scientific, cat. number 32106) or Clarity Western ECL Substrate (Bio-Rad, cat. number 1705061) prior to detection with the iBright™ CL1500 Imaging System (Thermo Fisher Scientific, cat. number A44240).

### Antibody screening by immunoprecipitation

Antibody-bead conjugates were prepared by adding 2 μg to 500 μl of Pierce IP Lysis Buffer from Thermo Fisher Scientific (cat. number 87788) in a microcentrifuge tube, together with 30 μl of Dynabeads protein A- (for rabbit antibodies) or protein G- (for mouse antibodies) (Thermo Fisher Scientific, cat. number 10002D and 10004D, respectively). All tubes were rocked for ~1 h at 4°C followed by two washes to remove unbound antibodies.

HAP1 WT lysates were collected in Pierce IP buffer (25 mM Tris-HCl pH 7.4, 150 mM NaCl, 1 mM EDTA, 1% NP-40 and 5% glycerol) supplemented with protease inhibitor. Lysates were rocked 30 min at 4°C and spun at 110,000 *x g* for 15 min at 4°C. 0.5 ml aliquots at 1 mg/ml of lysate were incubated with an antibody-bead conjugate for 1 h at 4°C. The unbound fractions were collected, and beads were subsequently washed three times with 1.0 ml of IP buffer and processed for SDS-PAGE and western blot on precast midi 4–20% Tris-Glycine polyacrylamide gels.

## Data Availability

Zenodo: Dataset for the ARID1B antibody screening study **https://doi.org/10.5281/zenodo.19069583** **.** ^11^ Data are available under the terms of the Creative Commons Attribution 4.0 International license (CC-BY 4.0).
