# Supplementary material for: A guide to selecting high-performing antibodies for ARID1B (UniProt ID: Q8NFD5) for use in western blot and immunoprecipitation
Source: F1000Res. 2026 Apr 28;15:631. [Version 1] doi: 10.12688/f1000research.179398.1 (PMC13616055; doi:10.12688/f1000research.179398.1)
Supplement: Peer review history

# Reviewer report: Reviewer response for version 1

**DOI:** 10.5256/f1000research.197904.r518765

**Published:** 2026-09-26

**Authors:** Deborah Moshinsky

**Affiliations:** 1 Institute for Protein Innovation, Boston, Massachusetts, USA

ARID1B plays a critical role in transcriptional regulation, cell differentiation, and development as a key component of the SWI/SNF (BAF) chromatin remodeling complex.  The authors tested 7 commercially available antibodies for their ability to detect ARID1B protein in Western Blot and Immunoprecipitation using standardized protocols as part of a larger initiative to provide open-source antibody characterization data.  The protocols are well described, and the results are clearly shown.  The authors explain that they are not experts in the biology or interpretation of the results but that they are providing it so scientists can have more data to make useful conclusions for their own biological studies where antibody validation data forms an important basis.  Antibodies that were apparently successful in Western Blot (in wt vs. KO cells) and immunoprecipitation were found for the target. 

 Note that an inappropriate reference to IF appears where it says immunoprecipitation is also referred to as immunocytochemistry, and the authors are encouraged to remove that wording.  Additionally, in Figure 4, the Western blot cartoon with bands for the wt and knockout samples says "target not detected" underneath but would be more accurately described as 'target not necessarily detected' or 'target binding not established' or something similar.

Are sufficient details of methods and materials provided to allow replication by others?

Yes

Is the rationale for creating the dataset(s) clearly described?

Yes

Are the datasets clearly presented in a useable and accessible format?

Yes

Are the protocols appropriate and is the work technically sound?

Yes

Reviewer Expertise:

Antibody characterization and validation

I confirm that I have read this submission and believe that I have an appropriate level of expertise to confirm that it is of an acceptable scientific standard.

# Reviewer report: Reviewer response for version 1

**DOI:** 10.5256/f1000research.197904.r512463

**Published:** 2026-09-08

**Authors:** Steve Jean

**Affiliations:** 1 University of Sherbrooke, Sherbrooke, Québec, Canada

This data note evaluates seven different renewable antibodies against ARID1B using western blotting and immunoprecipitation. The manuscript is part of the broader YCharOS initiative, which aims to characterize antibodies from a range of vendors using standardized protocols. The manuscript is clearly written, and the data are adequately presented. The fact that the original images are directly accessible through Zenodo is also a strength. The authors appropriately highlight the limitations of their study, and the use of two independent ARID1B knockout cell lines further strengthen the work.

 I have two points that could improve the presentation and interpretation of the data.

 First, it would be helpful to indicate in the Methods section whether different exposure times were used for the antibodies shown in Figures 1 and 2. This information would provide the community with an additional parameter for evaluating antibody performance and could be included in the figure legend.

 Second, the same amount of antibody (2 µg) was used for all co-immunoprecipitation experiments, yet for some antibodies (65747, 92964, and MABN2266), the heavy chain is very difficult to see in the provided Ponceau images. This makes interpretation of the data more challenging, as an apparent lack of immunoprecipitation could potentially result from a low amount of antibody bound to the beads. If the authors could provide an explanation for this observation, or acknowledge it as a limitation, it would strengthen the manuscript.

 Finally, there are one reference to immunofluorescence (IF) in the text, but no IF data are presented in the manuscript. Therefore, I would suggest removing the discussion of IF from the limitations section.

Are sufficient details of methods and materials provided to allow replication by others?

Yes

Is the rationale for creating the dataset(s) clearly described?

Yes

Are the datasets clearly presented in a useable and accessible format?

Yes

Are the protocols appropriate and is the work technically sound?

Yes

Reviewer Expertise:

Membrane trafficking, proteomics

I confirm that I have read this submission and believe that I have an appropriate level of expertise to confirm that it is of an acceptable scientific standard.
